# The impact of advanced maternal age on the risk of perineal and anal sphincter injuries in nulliparous women

**DOI:** 10.1002/ijgo.71003

**Published:** 2026-04-10

**Authors:** Omri Dominsky, Emmanuel Attali, Maayan Maor, Ronen Gold, Asnat Groutz, Yariv Yogev, Yoav Baruch

**Affiliations:** ^1^ Department of Obstetrics and Gynecology Lis Hospital for Women's Health, Tel Aviv Sourasky Medical Center Tel Aviv Israel; ^2^ Gray Faculty of Medical and Health Sciences Tel Aviv University Tel Aviv Israel

**Keywords:** advanced maternal age, obstetric anal sphincter injury, perineal injury, primiparous

## Abstract

**Objective:**

The aim of the present study was to determine whether advanced maternal age is associated with increased risk of perineal injury and obstetric anal sphincter injury (OASI) among primiparous women.

**Methods:**

This was a retrospective cohort study of primiparous women aged 20–50 years with singleton term pregnancies in cephalic presentation who delivered vaginally at a single tertiary center between 2012 and 2024. Women were stratified into three groups: 20–30 (reference), 30–40, and 40–50 years. The primary outcome was overall perineal injury, defined as spontaneous perineal laceration, labial tear, episiotomy, or OASI. The secondary outcome was OASI, analyzed separately. Multivariable logistic regression assessed the association between maternal age and each outcome.

**Results:**

Among 45 021 primiparous women, 18 230 (40.5%) were aged 20–30 years, 25 520 (56.7%) aged 30–40 years, and 1271 (2.8%) aged 40–50 years. Overall perineal injury rates were similar across groups (86.0%, 85.0%, and 85.3%), and the only significant pairwise difference was between women aged 20–30 and 30–40 years (*P* = 0.008). OASI rates were consistently low (0.8%, 0.9%, and 0.7%; *P* = 0.589). In multivariable analysis, women aged 30–40 years had lower odds of overall perineal injury compared with the reference group (adjusted odds ratio [aOR] 0.89; 95% confidence interval [CI]: 0.84–0.95; *P* < 0.001), whereas women aged 40–50 years showed no significant difference (aOR 0.92; 95% CI: 0.76–1.11; *P* = 0.371). In the model for the secondary outcome, maternal age was not associated with OASI.

**Conclusion:**

Advanced maternal age is not an independent predictor of perineal injury or OASI. Clinical counseling should emphasize modifiable risk factors rather than maternal age.

## 1 INTRODUCTION

Perineal injury is one of the most common morbidities of vaginal birth, affecting up to 90% of primiparous women and ranging from minor first‐degree lacerations to obstetric anal sphincter injuries (OASI).^1^, ^2^ Among primiparous women, OASI occurs in 1%–3% of deliveries and carries risk of persistent morbidity including anal incontinence, dyspareunia, and chronic pain.^3^, ^4^, ^5^

Over the past three decades, maternal age at first birth has risen steadily worldwide, particularly in high income countries, where women aged 35 years and older now account for an increasing proportion of first deliveries.^6^, ^7^ This demographic shift has raised concerns regarding a potential association between advancing maternal age and perineal injury. Previous studies examining this association have reported inconsistent findings. A registry analysis of nearly one million births reported progressively increasing OASI rates with maternal age,^8^ a finding corroborated by two large Swedish registry studies.^9^, ^10^ In contrast, other cohort studies found no independent association after adjustment for obstetric factors, with operative delivery and birth weight emerging as the dominant predictors.^11^, ^12^ Many of these studies relied on large registry‐based data from multiple centers with heterogeneous obstetric practices, and commonly dichotomized maternal age at 35 years. Such approaches may obscure potential nonlinear associations across the reproductive lifespan and limit the ability to evaluate older maternal age groups in greater detail. In particular, women in the fifth decade of life represent a distinct and increasingly relevant obstetric population that has rarely been examined separately.

Accordingly, the present study aimed to evaluate the association between maternal age and perineal injury across three clinically meaningful age groups within a large single‐center cohort with standardized labor management protocols, allowing more consistent assessment of the independent contribution of maternal age to perineal injury risk.

## 2 MATERIALS AND METHODS

This was a retrospective cohort study conducted at a single, tertiary, university‐affiliated medical center, with approximately 13 000 deliveries annually between January 2012 and January 2024.

The study was approved by the local Institutional Review Board (approval no.: TLVMC‐0221‐25). The requirement for informed consent was waived due to the retrospective design, and all data were anonymized prior to analysis.

### 2.1 Study population

Eligible participants were primiparous women aged 20–50 years with a singleton term pregnancy (≥37 weeks) in cephalic presentation who delivered vaginally at our institution. The patient selection process is illustrated in Figure 1.

**FIGURE 1:**
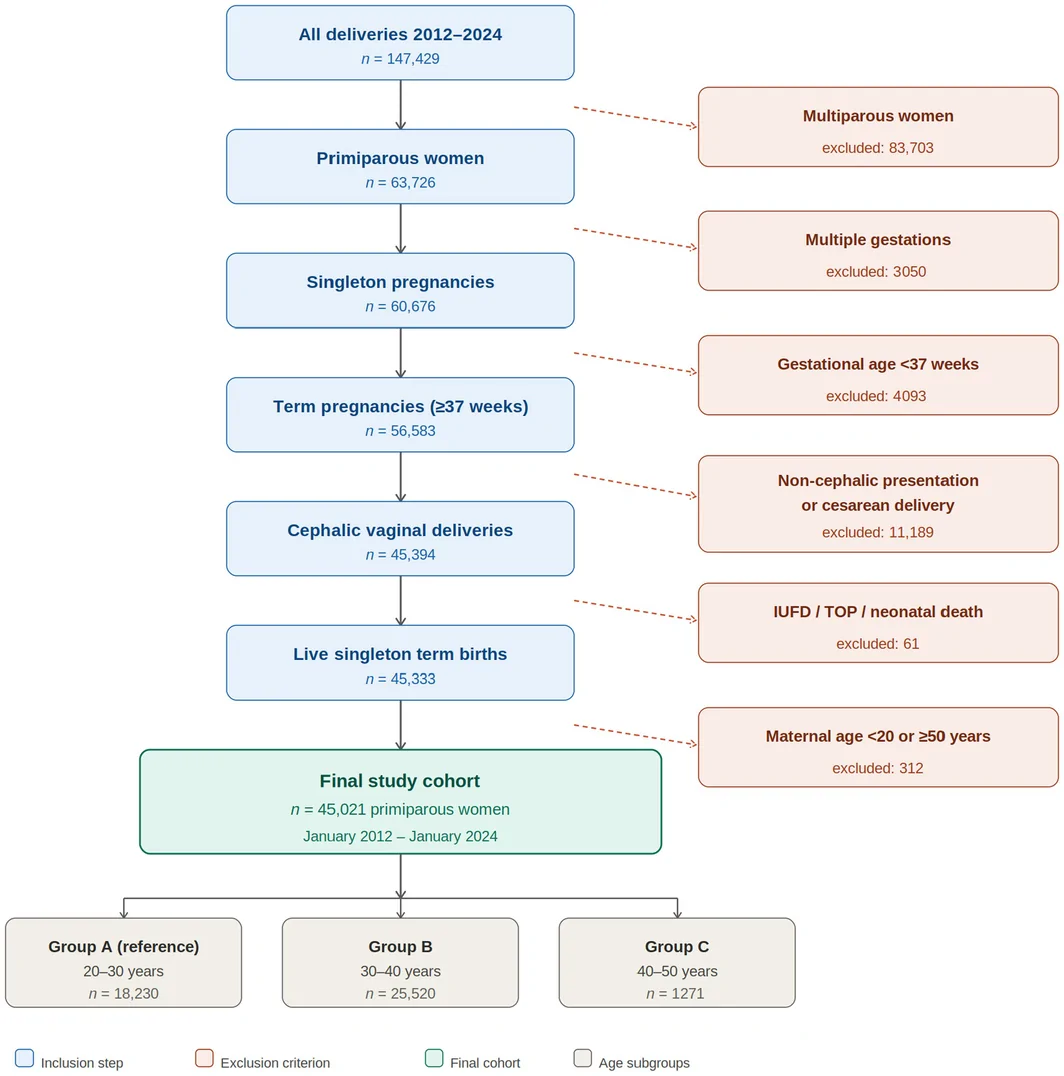
Patient selection flow chart.

Maternal age was categorized into three clinically meaningful groups: 20–30 years (group A, reference), 30–40 years (group B), and 40–50 years (group C), to evaluate perineal injury risk across the reproductive lifespan with particular focus on women in the fifth decade.

### 2.2 Data collection and study definitions

A standardized classification system for perineal lacerations was applied consistently throughout the study period. All data were retrospectively extracted from comprehensive electronic medical records. Data collected included maternal demographics, intrapartum variables, and obstetrical and neonatal outcomes. Data regarding spontaneous perineal tears, need for perineal repair, episiotomyrates, OASI, length of hospital stay after delivery, and need for blood transfusion were also recorded. Maternal age, body mass index (BMI, calculated as weight in kilograms divided by the square of height in meters), gestational age at delivery, induction of labor, prolonged second stage, analgesia type, episiotomy, and vacuum‐assisted delivery were considered potential confounders that may have influenced the rate of perineal tears.

Prolonged second stage was defined as a duration exceeding 3 h in women with epidural analgesia and 2 h in those without. Episiotomies in our institution are mediolateral and performed selectively, at the discretion of the attending midwife or obstetrician, particularly in cases of operative vaginal delivery, which in our institution are performed by vacuum extraction only.

Perineal tears were classified according to the Royal College of Obstetricians and Gynecologists Green‐top Guideline^13^: first‐degree lacerations involve the perineal skin and vaginal mucosa only. Second‐degree lacerations extend into the perineal muscles but not the anal sphincter. Third‐degree lacerations involve the anal sphincter complex. Fourth‐degree lacerations extend through the anal sphincter complex into the rectal mucosa.

The primary outcome was overall perineal injury, defined as a composite outcome including spontaneous perineal lacerations, labial tears, episiotomy, or obstetric anal sphincter injury (OASI). The secondary outcome was OASI, analyzed separately as an individual component of the composite.

### 2.3 Statistical analysis

Categorical variables are presented as frequencies and percentages. Continuous variables were assessed for normality using visual inspection of histograms and Q–Q plots, and are presented as mean ± standard deviation (SD) or median with interquartile range accordingly. Categorical variables were compared using the Chi‐square test, normally distributed continuous variables using one‐way analysis of variance (ANOVA), and non‐normally distributed variables using the Kruskal–Wallis test. Multivariable logistic regression was performed to examine the association between maternal age and perineal injury, adjusting for gestational age, ethnicity, gestational diabetes, smoking, induction of labor, epidural analgesia, prolonged second stage, vacuum‐assisted delivery, and birth weight. To account for potential temporal bias, year of delivery was included as a covariate in both models. As a sensitivity analysis, maternal age was also categorized into 5‐year intervals (<25, 25–29, 30–34, 35–39, and ≥40 years) and entered into the multivariable models for both outcomes. The results are presented in Table S1. Age and perineal injury data were available for all participants. The proportion of missing data for other covariates was minimal and no imputation was performed. All statistical tests were two‐sided with a significance threshold of *P* less than 0.05. Analyses were conducted using SPSS software (version 29.0.2; IBM Corporation, Armonk, New York, USA).

## 3 RESULTS

Overall, 45 021 primiparous women met the inclusion criteria. Of these, 18 230 (40.5%) were aged 20–30 years (group A), 25 520 (56.7%) were aged 30–40 years (group B), and 1271 (2.8%) were aged 40–50 years (group C), with corresponding mean ages of 26.9 ± 2.6, 33.3 ± 2.5, and 41.8 ± 1.6 years, respectively.

Baseline maternal demographics, pregnancy characteristics, and intrapartum factors are presented in Table 1. The prevalence of gestational diabetes (7.7%, 11.3%, and 17.7%) and the use of fertility treatments (5.5%, 14.8%, and 59.8%) increased substantially with advancing maternal age (all *P* < 0.001). Rates of labor induction (20.1%, 21.5%, and 28.9%), meconium‐stained amniotic fluid (18.7%, 21.3%, and 23.6%), non‐reassuring fetal heart rate (14.8%, 19.4%, and 24.1%), and prolonged second stage of labor (11.5%, 15.1%, and 18.7%) all increased significantly with advancing maternal age (*P* < 0.001 for all). Vacuum‐assisted delivery also increased across age categories (15.8%, 21.9%, and 28.7%; *P* < 0.001). Mean birth weight declined progressively with advancing maternal age (3245 ± 390 g, 3205 ± 391 g, and 3154 ± 398 g; *P* < 0.001).

**TABLE 1:** Baseline, intrapartum, and delivery characteristics by maternal age group.

Characteristic	20–30 years (*n* = 18 230) 40.5%	30–40 years (*n* = 25 520) 56.7%	40–50 years (*n* = 1271) 2.8%	*P* value
*Maternal demographics*				
Maternal age, years (mean ± SD)	26.9 ± 2.6	33.3 ± 2.5	41.8 ± 1.6	–
Pre‐pregnancy BMI (median, IQR)	21.4 (19.5–23.7)	21.4 (19.7–23.6)	22.1 (20.2–24.9)	<0.001^b,c^
Ethnicity				0.042
Caucasian, *n* (%)	17 395 (95.5)	24 678 (96.7)	1221 (96.1)	a
Asian, *n* (%)	291 (1.6)	576 (2.3)	30 (2.4)	a,b
African, *n* (%)	530 (2.9)	253 (1.0)	20 (1.6)	a,b,c
Smoking during pregnancy, *n* (%)	648 (3.7)	807 (3.3)	71 (6.0)	<0.001^a,b,c^
*Pregnancy characteristics*				
Gestational diabetes, *n* (%)	1395 (7.7)	2873 (11.3)	225 (17.7)	<0.001^a,b,c^
Fertility treatment, *n* (%)	964 (5.5)	6903 (14.8)	730 (59.8)	<0.001^a,b,c^
Gestational age at delivery, weeks (mean ± SD)	39.8 ± 1.1	39.8 ± 1.1	39.7 ± 1.2	<0.001^b,c^
*Labor characteristics*				
Labor induction, *n* (%)	3628 (20.1)	5423 (21.5)	363 (28.9)	<0.001^a,b,c^
Epidural analgesia, *n* (%)	15 165 (83.2)	20 484 (80.3)	1039 (81.7)	<0.001
Prolonged second stage, *n* (%)	2020 (11.5)	3718 (15.1)	229 (18.7)	<0.001^a,b,c^
Occiput posterior, *n* (%)	584 (3.2)	870 (3.4)	38 (3.0)	0.403
Meconium‐stained amniotic fluid, *n* (%)	3404 (18.7)	5420 (21.3)	299 (23.6)	<0.001^a,b,c^
Non‐reassuring fetal heart rate, *n* (%)	2696 (14.8)	4945 (19.4)	306 (24.1)	<0.001^a,b,c^
*Delivery characteristics*				
Vacuum‐assisted delivery, *n* (%)	2872 (15.8)	5594 (21.9)	365 (28.7)	<0.001^a,b,c^
Birth weight, grams (mean ± SD)	3245 ± 390	3205 ± 391	3154 ± 398	<0.001^a,b,c^

*Note*: BMI, calculated as weight in kilograms divided by the square of height in meters. Superscripts indicate statistically significant differences: ^a^: Indicates significant difference between 20–30 and 30–40 years, ^b^: between 20–30 and 40–50 years, ^c^: between 30–40 and 40–50 years. Normally distributed continuous variables were compared using ANOVA, non‐normally distributed continuous variables were compared using the Kruskal–Wallis test.

Abbreviations: ANOVA, one‐way analysis of variance; BMI, body mass index; IQR, interquartile range; SD, standard deviation.

Overall perineal injury rates remained similar across maternal age groups: 86.0%, 85.0%, and 85.3% in the 20–30, 30–40, and 40–50‐year age groups, respectively, with statistical significance observed only between groups A and B (*P* = 0.008) (Table 2). OASI rates remained low across all age groups (0.8%, 0.9%, and 0.7%; *P* = 0.589). Episiotomy rates were similar in groups A and B but higher among women aged 40–50 years (36.6%, 36.2%, and 40.9%; *P* = 0.003). Additional adverse maternal outcomes that increased with advancing age included a greater reduction in mean hemoglobin level, higher rates of packed red blood cell transfusion, and prolonged hospital stay (Table 2, all *P* < 0.001).

**TABLE 2:** Perineal injury and maternal outcomes by age group.

Outcome	20–30 years (*n* = 18 230)	30–40 years (*n* = 25 520)	40–50 years (*n* = 1271)	*P* value
*Primary outcome*				
Overall perineal injury, *n* (%)	15 682 (86.0)	21 680 (85.0)	1084 (85.3)	0.008^a^
Spontaneous perineal laceration, *n* (%)	10 068 (55.2)	13 655 (53.5)	644 (50.7)	<0.001^a,b,c^
Spontaneous labial tears, *n* (%)	1835 (10.1)	2450 (9.6)	99 (7.8)	0.002^b,c^
Episiotomy, *n* (%)	6671 (36.6)	9247 (36.2)	520 (40.9)	0.003^b,c^
*Secondary outcomes*				
OASI, *n* (%)	149 (0.8)	222 (0.9)	8 (0.6)	0.589
Third‐degree tears, *n* (% of OASI)	135 (90.6)	212 (95.5)	8 (100.0)	0.126
Fourth‐degree tears, *n* (% of OASI)	14 (9.4)	10 (4.5)	0 (0.0)	0.126
*Additional maternal outcomes*				
Hemoglobin change, g/dL (mean ± SD)	−2.54 ± 1.6	−2.78 ± 1.7	−3.28 ± 1.8	<0.001^a,b,c^
Packed red blood cell transfusion, *n* (%)	331 (1.8)	567 (2.2)	57 (4.5)	<0.001^a,b,c^
Prolonged length of stay (>3 days), *n* (%)	2860 (15.7)	3805 (17.9)	278 (21.9)	<0.001^a,b,c^

*Note*: Superscripts indicate statistically significant differences: ^a^: Indicates significant difference between 20–30 and 30–40 years; ^b^: between 20–30 and 40–50 years; ^c^: between 30–40 and 40–50 years.

Abbreviations: OASI, obstetric anal sphincter injury; SD, standard deviation.

In multivariable analysis, women aged 30–40 years had lower odds of overall perineal injury compared with women aged 20–30 years (adjusted odds ratio [aOR] 0.89, 95% confidence interval [CI]: 0.84–0.95), whereas women aged 40–50 years did not differ significantly from the reference group (aOR 0.92; 95% CI: 0.76–1.11; *P* = 0.371) (Table 3). When maternal age was modeled as a continuous variable, each one‐year increase in age was associated with lower odds of overall perineal injury (aOR 0.98; 95% CI: 0.97–0.99; *P* < 0.001). Maternal age was not significantly associated with OASI risk. Vacuum‐assisted delivery was the strongest predictor of overall perineal injury (aOR 3.15), whereas Asian ethnicity showed a strong association with OASI (aOR 4.46). Year of delivery was independently associated with overall perineal injury (aOR 1.16; 95% CI: 1.15–1.17; *P* < 0.001) but not with OASI (aOR 1.01; 95% CI: 0.98–1.04; *P* = 0.591).

**TABLE 3:** Multivariable logistic regression models for factors associated with perineal and anal sphincter injury.

Variable	Overall perineal injury		OASI	
	aOR (95% CI)	*P* value	aOR (95% CI)	*P*‐value
*Maternal age*				
20–30 years (reference)	1.000	–	1.000	–
30–40 years	0.893 (0.843–0.950)	<0.001	0.904 (0.720–1.135)	0.383
40–50 years	0.916 (0.757–1.110)	0.371	0.624 (0.273–1.425)	0.263
Continuous (per 1‐year increase)^a^	0.980 (0.97–0.990)	<0.001	0.978 (0.953–1.004)	0.101
*Ethnicity*				
Caucasian (reference)	1.000	–	1.000	–
Asian	1.617 (1.215–2.152)	0.001	4.457 (2.855–6.958)	<0.001
African	1.213 (0.851–1.728)	0.286	0.795 (0.196–3.219)	0.748
*Pregnancy and labor factors*				
Smoking during pregnancy	0.652 (0.566–0.750)	<0.001	0.412 (0.153–1.108)	0.079
Gestational diabetes	1.070 (0.963–1.189)	0.210	1.131 (0.792–1.614)	0.498
Gestational age (weeks)	1.065 (1.034–1.097)	<0.001	0.934 (0.833–1.046)	0.236
Induction of labor	1.253 (1.156–1.359)	<0.001	0.896 (0.675–1.190)	0.449
Epidural analgesia	1.489 (1.382–1.603)	<0.001	0.574 (0.433–0.761)	<0.001
Prolonged second stage	1.004 (0.907–1.111)	0.942	1.383 (1.042–1.835)	0.025
Vacuum‐assisted delivery	3.151 (2.824–3.517)	<0.001	1.829 (1.414–2.366)	<0.001
Birth weight (per 100 g increase)	1.066 (1.056–1.076)	<0.001	1.121 (1.087–1.156)	<0.001
Year of delivery	1.16 (1.15–1.17)	<0.001	1.01 (0.98–1.04)	0.591

*Note*: Caucasian ethnicity was used as the reference category because it represented the largest and most stable demographic group.

Abbreviations: aOR, adjusted odds ratio; CI, confidence interval; OASI, obstetric anal sphincter injury.

^a^ Maternal age was additionally modeled as a continuous variable in a separate multivariable logistic regression model adjusted for the same covariates.

## 4 DISCUSSION

In this cohort of 45 021 primiparous women, the main findings were as follows:

(1) Maternal age in the fifth decade (40–50 years) was not associated with increased risk of overall perineal injury or OASI. (2) Women aged 30–40 years had lower odds of overall perineal injury compared with women aged 20–30 years. (3) OASI rates remained consistently low across all age groups (0.6%–0.9%). (4) Vacuum‐assisted delivery was the strongest predictor for overall perineal injury (aOR 3.15).

### 4.1 Results in the context of previous studies

Our findings differ from several large population‐based studies. The Swedish Medical Birth Register analysis of nearly one million births reported progressive increases in OASI rates with advancing maternal age, rising from 2.9% in women younger than 25 years to 4.6% in those older than 35 years.^8^ André et al. reported similar results in their 2022 Swedish registry study and prediction model, though the age‐risk relationship plateaued after 35 years.^9^, ^10^ In contrast, our findings are consistent with studies demonstrating no independent association between maternal age and OASI after adjustment for obstetric factors. Meister et al. found that maternal age was not a significant predictor of OASI in multivariable analysis, and Christianson et al. reported similar findings, with operative delivery and birth weight emerging as the strongest predictors in both studies.^11^, ^12^

These discrepancies likely reflect variations in obstetric practice across healthcare systems. Registry datasets aggregate outcomes from numerous hospitals with divergent practices, while single‐center studies with standardized protocols demonstrate different patterns. Our institution employs selective mediolateral episiotomy and standardized hands‐on perineal support during crowning, which may explain our exceptionally low OASI rates (0.6%–0.9%) and lack of age‐related increase.^14^, ^15^ Notably, our OASI‐specific analysis corroborates the substantial role of ethnicity, with Asian origin as the strongest independent predictor (aOR 4.46), as found by our recent study.^16^

### 4.2 Mechanisms and potential explanations

Although advancing maternal age is associated with physiological changes in pelvic floor structure including reduced tissue elasticity, lower collagen content, and diminished healing capacity,^17^, ^18^, ^19^ these alterations did not translate into higher perineal injury rates in our cohort. One explanation is the inverse association between maternal age and birth weight: women aged 40–50 years delivered smaller infants on average, which may reduce mechanical strain and offset age‐related tissue vulnerability.

Women in the older age groups demonstrated significantly higher rates of labor induction, prolonged second stage, and vacuum‐assisted delivery, consistent with previous reports.^8^, ^20^ Despite this intervention‐intensive profile, maternal age itself was not associated with increased perineal injury risk. Instead, specific intrapartum variables emerged as the principal determinants. Vacuum‐assisted delivery was the strongest predictor of overall perineal injury (aOR 3.15). This finding is consistent with extensive prior evidence highlighting the substantial contribution of operative vaginal birth to OASI risk.^16^, ^21^, ^22^ When examining OASI specifically, Asian ethnicity emerged as the strongest predictor (aOR 4.46). This association may reflect previously described differences in pelvic and perineal anatomy that could increase susceptibility to sphincter injury during vaginal birth.^16^ Labor induction was associated with a 25% increase in injury risk, and increasing birth weight demonstrated a clear dose–response relationship, reinforcing fetal size as a major mechanical contributor to perineal injury.^4^ Notably, while epidural analgesia increased overall perineal injury risk (aOR 1.49), it demonstrated a protective effect against OASI specifically (aOR 0.57), likely reflecting more controlled second stage management, as observed in our recent study.^23^ Episiotomy was more frequent in older women but did not increase OASI rates, reflecting selective use with standardized perineal protection.^15^

Smoking was associated with reduced odds of both outcomes, consistent with other reports.^24^, ^25^ The mechanism remains unclear but may involve alterations in tissue compliance. This finding should not diminish the importance of smoking cessation counseling but highlights the complex interplay between biological and mechanical factors in perineal injury pathophysiology.

The exceptionally low OASI rates observed in this cohort, ranging from 0.6% to 0.9%, remain well below the 1%–3% typically reported in contemporary literature for non‐instrumental deliveries.^3^, ^5^ The consistency of these low rates across all maternal age categories suggests that standardized obstetric practices may exert a more meaningful influence on perineal outcomes than the biological effects of maternal aging.

### 4.3 Strengths and limitations

The principal strength of this investigation is the large sample size of 45 021 primiparous women, which permitted robust analysis even among the relatively small subgroup of women aged 40–50 years. The utilization of a comprehensive single‐center electronic medical records system ensured data consistency and minimized measurement bias.

Several limitations warrant consideration. The single‐center design may limit generalizability, as our institutional protocols and the predominantly middle‐to‐high socioeconomic population served by our institution, composed largely of Caucasian women, may not reflect global obstetric care patterns. Selection bias was inherent to the study design, as older and higher‐risk women were more frequently delivered by cesarean section and consequently excluded from analysis. Future prospective investigations examining long‐term functional outcomes of perineal injury across different maternal age groups would provide valuable insights into the clinical significance of age‐related injury patterns. Stratified analyses by delivery mode and formal sensitivity analyses were not performed and represent areas for future investigation.

Another limitation is the potential for temporal bias, as obstetric practices such as episiotomy use, operative delivery rates, and recognition of OASI may evolve over time. Although our institution follows standardized protocols for perineal injury classification throughout the study period, residual temporal variation cannot be excluded. To partially address this, year of delivery was included as a covariate in the multivariable models. Year of delivery was independently associated with overall perineal injury but not with OASI, suggesting a modest increase in overall perineal injury rates over the study period, likely reflecting evolving obstetric practices rather than a change in OASI risk. To address the potential dilution of age‐related trends within broad 10‐year categories, a supplementary analysis using 5‐year age intervals was performed and confirmed the absence of an adverse age‐related effect on perineal injury risk.

## 5 CONCLUSION

In this large cohort of primiparous women, advancing maternal age was not associated with an increased risk of perineal injury, including OASI. These findings suggest that clinical counseling should emphasize evidence‐based prevention strategies, rather than maternal age alone, as the key to reducing perineal injury.

## AUTHOR CONTRIBUTIONS

**Omri Dominsky:** Writing—original draft. **Emmanuel Attali:** Investigation, writing—review and editing. **Maayan Maor:** Writing—review and editing. **Ronen Gold:** Writing—review and editing. **Asnat Groutz:** Writing—review and editing. **Yariv Yogev:** Writing—validation, review and editing, supervision. **Yoav Baruch:** Conceptualization, data curation and analysis, writing—original draft, project administration. All authors revised the article for important intellectual content and approved the final version submitted for publication.

## FUNDING INFORMATION

This research did not receive any specific grant from funding agencies in the public, commercial, or not‐for‐profit sectors.

## CONFLICT OF INTEREST STATEMENT

The authors declare no conflict of interest.

## Supporting information


**Table S1.** Multivariable logistic regression for overall perineal injury and OASI using 5‐year maternal age categories.

## Data Availability

The data are available from the corresponding author upon reasonable request.
