# Supplementary material for: The impact of advanced maternal age on the risk of perineal and anal sphincter injuries in nulliparous women
Source: Int J Gynaecol Obstet. 2026 Apr 10;175(1):207–14. doi: 10.1002/ijgo.71003 (PMC13629572; doi:10.1002/ijgo.71003)
Supplement: Supplementary file 1 [file IJGO-175-207-s001.docx]

| **Supplementary Table S1. Multivariable logistic regression for overall perineal injury and OASI using 5-year maternal age categories** | | | | |
| --- | --- | --- | --- | --- |
| **Variable** | **Overall Perineal Injury** | | **OASI** | |
|  | **aOR (95% CI)** | **P-value** | **aOR (95% CI)** | **P-value** |
| **MATERNAL AGE** | | | | |
| <25 years (reference) | 1.000 | – | 1.000 | – |
| 25-29 years | 0.790 (0.690–0.890) | <0.001 | 0.780 (0.520–1.170) | 0.227 |
| 30-34 years | 0.730 (0.650–0.830) | <0.001 | 0.740 (0.500–1.100) | 0.139 |
| 35-39 years | 0.630 (0.550–0.730) | <0.001 | 0.730 (0.460–1.170) | 0.191 |
| ≥40 years | 0.740 (0.600–0.930) | <0.001 | 0.510 (0.210–1.230) | 0.136 |

Models adjusted for ethnicity, smoking, gestational diabetes, gestational age, induction of labor, epidural analgesia, prolonged second stage, vacuum-assisted delivery, birthweight, and year of delivery. aOR, adjusted odds ratio; CI, confidence interval; OASI, obstetric anal sphincter injury.
